# Changes in anisometropia by age in children with hyperopia, myopia, and antimetropia

**DOI:** 10.1038/s41598-023-40831-0

**Published:** 2023-08-22

**Authors:** Han-Wen Lin, Meng-Ling Young, Christy Pu, Chung-Ying Huang, Ken-Kuo Lin, Jiahn-Shing Lee, Chiun-Ho Hou

**Affiliations:** 1 Department of Dermatology, Chang Gung Memorial Hospital, Linkou, Taiwan; 2 Department of Medicine, School of Medicine, Chang Gung University, Taoyüan, Taiwan; 3 Department of Ophthalmology, Chang Gung Memorial Hospital, Linkou, Taiwan; 4 Institute of Public Health, School of Medicine, National Yang Ming Chiao Tung University, Taipei, Taiwan; 5 Department of Ophthalmology, National Taiwan University Hospital, 7, Chung Shan S. Rd., Zhongzheng Dist., Taipei, 100225 Taiwan (R.O.C.)

**Keywords:** Medical research, Epidemiology

## Abstract

Anisometropia is a unique condition of both eyes and it is associated with vision problems such as amblyopia and reduced stereoacuity. Previous studies have not reported its change pattern by age and its correlation with the refractive condition of both eyes. This study aims to compare the changes in anisometropia by age in children with hyperopia, myopia, and antimetropia. In total, 156 children were included. Children aged 3–11 years with anisometropia ≥ 1.00 D were followed up for ≥ 1 year with ≥ 2 visits at two medical centers in Taiwan. Refractive errors by cycloplegic autorefractometry, best-corrected visual acuity, eye position, and atropine use were recorded. The children were divided into hyperopic, myopic, and antimetropic groups. The results showed that anisometropia decreased in children aged < 6 years (3.34–2.96 D; P = 0.038) and increased in older children (2.16–2.55 D; P = 0.005). In children aged 3, 4, 5, and 6 years, the mean anisometropia was higher in children with myopia and antimetropia than in those with hyperopia (P = 0.005, 0.002, 0.001, and 0.011, respectively). The differences were not significant in children aged > 6 years (all P > 0.05). The factors associated with changes in anisometropia were age, refractive group, amblyopia, and strabismus. Anisometropia decreased with age in children younger than 6 years, and the changes in anisometropia was found in children with myopia and antimetropia.

## Introduction

Anisometropia is a unique condition in which the 2 eyes of an individual differ in refractive error by ≥ 1.0 D. Studies have reported its prevalence to range from 1.27 to 7% in different age and ethnic groups^1–4^. Moreover, it is associated with vision problems, such as amblyopia and stereoacuity^5^. Similar to the wide range of distribution of refraction in neonates, infantile anisometropia results from the stochastic combination of axial length and ocular refractive components in both eyes^6^. As children grow, some infantile anisometropia undergoes a change in magnitude through emmetropization^7^, resulting in emmetropia or low hyperopia; the process is largely completed by the age of 6 years^6^. Therefore, age is a crucial factor that affects changes in anisometropia.

Several studies have investigated the changes in anisometropia that occur with age in children^1,7–13^; of these, some included children going through the emmetropization process. The prevalence of anisometropia decreased in a US-based cohort study, which measured the condition by using noncycloplegic retinoscopy^1^. In a Sweden-based study of 20 children, anisometropia decreased in the majority of the children^7^. Two studies focused on children with myopia^12,13^. Of these, the Egypt-based study reported that anisometropia increased until the age of 2 years and was followed by a nonsignificant decrease^12^; the Italy-based study reported that anisometropia decreased with age^13^. Another Taiwan-based study on amblyopic children demonstrated that anisometropia decreased in children with myopia but remained the same in children with hyperopia^8^.

Although much research has been conducted on anisometropia, 2 gaps remain. First, the changes in anisometropia and its correlation with age remain unclear. Second, whether the different changes between refractive groups also occur in children without amblyopia remains unclear. Therefore, we investigated the changes in anisometropia in Taiwanese children in different refractive groups and ages, and identified the associated factors.

## Methods

This retrospective study reviewed the medical charts of children with anisometropia presenting to the ophthalmology clinic at Linkou and Taipei Chang Gung Memorial Hospitals in Taiwan for consultant with 1 of 3 ophthalmologists (MLY, CYH, and CHH) between April 2018 and December 2018. Children aged 3–11 years and presenting for the first time were included. Anisometropia was defined as a spherical equivalent (SE) difference of ≥ 1.00 D, as suggested by previous studies^1,9^. To reveal a trend in changes in anisometropia, children who were followed up for at least a year with a minimum of 2 visits were included. We excluded children with organic ocular disorders, such as corneal opacity, congenital cataract, congenital malformation, or congenital glaucoma. The study was conducted in accordance with the tenets of the Declaration of Helsinki. The Institutional Review Board of Chang Gung Memorial Hospital in Linkou, Taiwan approved the study protocol (IRB number: 202001367B0), and the Institutional Review Board of Chang Gung Memorial Hospital in Linkou, Taiwan waived the requirement of written informed consent.

The information on demographics, refractive error, strabismus, amblyopia, best corrected visual acuity, and atropine use (in one or both eyes for at least 1 year) was retrieved from medical records. The refractive errors were obtained using cycloplegic autokeratorefractometry (Topcon KR-800, Topcon Corporation, Tokyo, Japan). Cycloplegia was achieved by applying 1% tropicamide twice, 20 min and 30 min before examination, or by atropine of 0.125%, 0.25% or 0.5% if the child was a regular user with nightly administration. Children were divided into 3 refractive groups according to the refractive conditions of both eyes, namely the hyperopic (both eyes SE > 0.00 D), antimetropic (one eye SE ≥ 0.00 D; the other eye ≤ 0.00 D), and myopic (both eyes SE < 0.00 D) groups, on the basis of the refraction of both eyes, as assessed at the first visit. We documented children whose refractive error change in one or both eyes during the follow-up period led to a change in their refractive group for at least 2 years.

Analysis of variance and chi-square tests were used to analyze differences in demographic characteristics for continuous and categorical variables, respectively. We used a paired *t* test to analyze the longitudinal changes in the SE difference. We also used analysis of variance (ANOVA) to perform an analysis of the between-group difference in the mean anisometropia among children in three refractive groups stratified by age, with the ages of the included children ranging from 3 to 13 years. We conducted an analysis of the proportions of children displaying varying degrees of anisometropia change from their first to last visits. For assessing the robustness of these results, we used both 0.00 D and 0.05 D as cutting points. In order to investigate which eye contributed to the changes in anisometropia, we categorized the two eyes of each child as the eye with a more positive SE, namely the eye with more hyperopia in the hyperopic and antimetropic groups or with less myopia in the myopic group, and the other eye with a less positive SE. Additionally, we used a generalized estimating equation (GEE) model with log link and Gaussian family accounting for clustered variance associated with repeated measurements of refractive error to identify factors associated with the changes in anisometropia. Baseline variables at the first visit, including refractive error, age, sex, amblyopia, strabismus, and atropine use for at least a year, were controlled in the anisometropia magnitude estimation. The statistical analyses were conducted using STATA 15 TX: StataCorp LLC^14^.

## Results

### Demographic characteristics

A total of 156 children were included in this study, with 50.6%, 26.9%, and 22.4% of them categorized into the hyperopic, myopic, and antimetropic groups, respectively (Table 1). The median age at the first visit was 5 years (range, 3–11 years), and the median follow-up period was 4 years (range, 1–10 years). The follow-up period was longer for children enrolled at the age of 3–5 years than children at the age of 6–11 years (4.80 vs. 3.16 years, P < 0.001). The mean initial anisometropia was 2.8 D (SD = 2.3 D). Moreover, 94 children with amblyopia and 15 children with high myopia (> 6.00 D in at least one eye) were identified at first visit. The sex distribution was similar in the 3 refractive groups. Age (*P* = 0.001) and anisometropia (*P* = 0.014) at the first visit differed among the 3 refractive groups. The number of children with amblyopia was higher in the hyperopic group at the first visit (*P* = 0.002).

**Table 1:** Demographic characteristics and spherical equivalent difference of children with anisometropia belonging to hyperopic, myopic, and antimetropic groups.

	Hyperopic	Myopic	Antimetropic	P-value
Number	79 (50.6%)	42 (26.9%)	35 (22.4%)	
Sex (Male/Female)^b^	42/37	20/22	19/16	0.803
First visit (years old)^a^				
Mean ± SD	5.2 ± 1.9	6.9 ± 2.6	5.8 ± 2.4	*0.001
Follow up years^a^				
Mean ± SD	4.4 ± 2.1	3.6 ± 2.4	3.8 ± 1.9	0.157
Spherical equivalent difference at first visit (Diopter)^a^				
Mean ± SD	2.29 ± 1.19	3.16 ± 3.22	3.53 ± 2.68	*0.014
Median	2.00	1.63	3.1	
Range	1.00 to 6.13	1.00 to 14.25	1.0 to13.0	
Spherical equivalent difference at last visit (Diopter)^a^				
Mean ± SD	2.30 ± 1.44	3.20 ± 3.19	3.34 ± 2.26	*0.028
Median	2.00	2.06	2.63	
Range	0.13 to 6.00	0.25 to 17.25	0.75 to 10.50	
Anisometropia change* from first to last visit (Diopter)^a^				
Mean ± SD	0.19 ± 1.41	0.04 ± 1.62	− 0.23 ± 1.59	0.672
Median	0.00	0.19	0.25	
Range	− 3.13 to 4.25	− 5.00 to 3.50	− 4.13 to 3.50	
Atropine use^b^				
Number	23 (29.1%)	31 (73.8%)	18 (51.4%)	* < 0.001
Amblyopia^b^				
First visit	58 (73.4%)	18 (42.9%)	18 (51.4%)	*0.002
Last visit	14 (17.7%)	10 (23.8%)	4 (11.4%)	0.369
Strabismus^b^				
No strabismus	39 (49.4%)	27 (64.3%)	19 (54.3%)	*< 0.001
Esotropia	30 (38.0%)	2 (4.7%)	3 (8.6%)	–
Exotropia	10 (12.7%)	13 (31.0%)	13 (37.1%)	–

SD, standard deviation.

^a^Analysis of variance for continuous variables, ^b^chi-square test for categorical variables, *anisometropia change is the differential refractive progression between the two eyes considering the positive or negative sign.

### Longitudinal changes in anisometropia in three refractive groups: younger versus older children

A nonsignificant decrease (by 0.22 D) in mean anisometropia was observed between the first and last visit in all children (*P* = 0.856). Because the age of 6 years is suggested as the emmetropization endpoint^6^, the children were divided into 2 groups based on their age. The percentage of children who experienced a reduction in anisometropia was higher in the group aged 3–5 years at enrollment (younger children) than in that aged 6–11 years at enrollment (older children). The reduction was determined using 0.00 D or 0.50 D as the cutoff (Table 2). Furthermore, between the first and last visits, anisometropia decreased significantly in younger children and increased significantly in older children. Among the younger children, the reduction of SE was greater in the eyes with more positive SE than in those with less positive SE (*P* = 0.038; Table 3). Among the older children, the reduction of SE was greater in the eyes with less positive SE than in those with more positive SE (*P* = 0.007).

**Table 2:** Anisometropia of children aged 3–5 years and 6–11 years at the first visit.

(a)				
First visit age (year)	Number	First anisometropia (D)	Last anisometropia (D)	p-value
3–5	86	3.34	2.96	*0.038
6–11	70	2.16	2.55	*0.005

(b)				
First visit age (year)	3–5	6–11	P-value	
0.00D as cut point				
Increased anisometropia	27	45	< 0.001	
Stable anisometropia	5	6		
Decreased anisometropia	54	19		
0.50D as cut point				
Increased anisometropia	18	31	< 0.001	
Stable anisometropia	27	25		
Decreased anisometropia	41	14		

D, diopter.

(a) Anisometropia difference between first and last visit. (b) Proportion of children with increased, stable, or decreased anisometropia between the first and last visit.

**Table 3:** The spherical equivalent changes in the two eyes of children with different age and refractive groups at the first visit.

Spherical equivalent changes between the last and first visit (mean ± SD)								
Age		3–5 years				6–11 years		
Two eyes of each child	Children number	Eye with more positive SE	Eye with less positive SE	P value	Children number	Eye with more positive SE	Eye with less positive SE	P value
All children	86	− 1.47 ± 0.15 D	− 1.09 ± 0.20 D	*0.038	70	− 1.18 ± 0.17 D	− 1.57 ± 0.17 D	*0.007
Hyperopic group	54	− 1.46 ± 0.18 D	− 1.13 ± 0.18 D	0.102	25	− 0.82 ± 0.18 D	− 1.48 ± 0.22 D	*0.010
Myopic group	15	− 2.14 ± 0.47 D	− 1.73 ± 0.77 D	0.487	27	− 1.61 ± 0.32 D	− 1.81 ± 0.35 D	0.308
Antimetropic group	17	− 0.87 ± 0.26 D	− 0.35 ± 0.40 D	0.263	18	− 1.00 ± 0.32 D	− 1.28 ± 0.20 D	0.417

Age, age at the first visit; D, diopter; SD , standard deviation; SE, spherical equivalent.

### Changes in anisometropia by age in the three refractive groups

**Figure 1:**
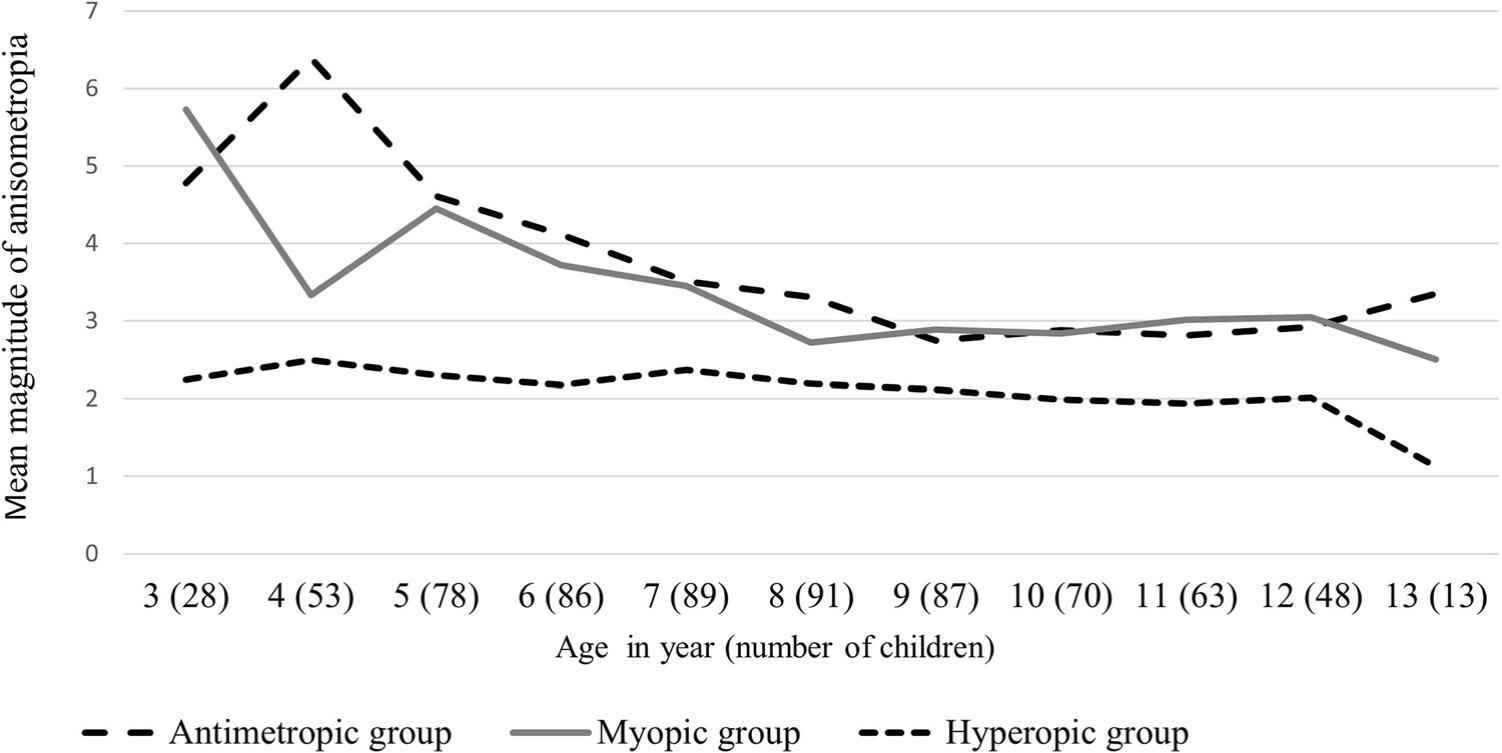
Mean anisometropia among the hyperopic, myopic, and antimetropic groups of children aged 3–13 years. The initial anisometropia had a higher magnitude in the myopic and antimetropic groups than in the hyperopic group. The mean anisometropia became lower in the myopic and antimetropic groups between the ages of 3 and 6 years, and the mean anisometropia was similar among the groups after the age of 6 years. The anisometropia in the hyperopic group remained similar between the ages of 3–13 years.

We calculated the mean anisometropia in the children, who were aged 3–13 years. The mean magnitude of anisometropia at different ages varied significantly among the three refractive groups (Fig. 1). The mean anisometropia in the myopic and antimetropic groups was higher in the children aged 3–6 years than that in the older age group, and the mean remained constant after the age of 6 years. The mean anisometropia among the children with hyperopia was similar across all age groups. In children aged 3–6 years, the mean anisometropia was higher in children with myopia and antimetropia than in those with hyperopia (ANOVA; *P* = 0.005, 0.002, 0.001, and 0.011 in children aged 3, 4, 5, and 6 years, respectively). However, the differences were no longer significant in children aged > 6 years (all *P* > 0.05).

### Factors associated with changes in anisometropia

In regression analysis, we controlled for possible confounding factors for changes in anisometropia, namely age, sex, amblyopia, refractive group, strabismus, and atropine use. Table 4 lists the SE difference estimated through GEE. The factors associated with changes in anisometropia were age, age square, refractive group, amblyopia, and strabismus. The correlation between changes in anisometropia and age does not exhibit linearity. The effect of age on anisometropia was negative for 1–9 years with a decrease in magnitude by age and became positive for age 10 and older. The antimetropic group also exhibited a higher anisometropia than the hyperopic group did (*P* < 0.001). Children with amblyopia or exotropia exhibited a higher anisometropia than those without amblyopia or exotropia (*P* = 0.034 and 0.027, respectively). Moreover, no association was observed between atropine use and anisometropia.

**Table 4:** Factors associated with anisometropia change.

Variable	Coefficient	Standard error	P > z	95% confidence interval
Age	− 0.225	0.0571	* < 0.001	− 0.337 to − 0.113
Age square	0.012	0.0031	* < 0.001	0.006 to 0.018
Amblyopia	0.578	0.2730	*0.034	0.043 to 1.114
Refraction group				
Hyperopia				
Myopia	0.337	0.1960	0.085	− 0.047 to 0.721
Antimetropia	0.817	0.1403	* < 0.001	0.542 to 1.092
Gender				
Male				
Female	0.172	0.2460	0.483	− 0.310 to 0.655
Strabismus				
No strabismus				
Esotropia	0.501	0.3148	0.111	− 0.116 to 1.118
Exotropia	0.683	0.3089	*0.027	0.078 to 1.288
Atropine use^a^	0.119	0.2668	0.657	− 0.404 to 0.642
Constant	1.590	0.5086	*0.002	2.205 to 3.053

^a^Atropine use is defined as use in one or both eyes for longer than 1 year.

## Discussion

In this study, we investigated the changes in anisometropia in 156 Taiwanese children with or without amblyopia and observed a significant decrease in anisometropia between the first and last visits among the children younger than 6 years at first visit and an increase in anisometropia in children at an older age. Even though the mean follow-up period was approximately 1.5 year longer for younger children than older children, the differences in the changes in anisometropia in these two age groups are unlikely the result of the shorter follow-up period used for the older children because the direction of changes in anisometropia was opposite. The changes in anisometropia by age were not similar among the children aged 3–13 years in the hyperopic, myopic, and antimetropic groups. The anisometropia is significant in younger children in the myopic and antimetropic groups and the differences in anisometropia by age became less notable from the age of 3–6 years; however, a relatively stable magnitude was observed in the hyperopic group. Moreover, the associated risk factors for changes in anisometropia were age, refractive group, amblyopia, and strabismus. Our study indicates that children of a younger age generally demonstrate a reduction in anisometropia as they age, and that the change in anisometropia is contingent upon their age and specific refractive conditions.

Our regression results reveal that the relationship between changes in anisometropia and age is not linear and anisometropia decreased the most among younger children. The findings are consistent with the current understanding of changes in refractive errors and the development of axial length and ocular refractive components in children undergoing emmetropization^6^. The principal determinants of refractive error in childhood are refraction at birth and the subsequent development of axial length and ocular refractive components with age. In a review study summarizing the refraction distribution and refractive error prevalence in childhood, the refractive error, including that in anisometropia, decreased by the age of 6 years^6^. Brown et al.^15^ reported that there was an abrupt reduction of hyperopia from infancy to the age of 3.5 years; the process continues at a slow rate until the age of 6 years. A previous study reported that the anisometropia prevalence declined from infancy to early childhood, followed by a rise until the age of 15 years^1^. The changes in anisometropia observed in our study support that a relationship is present between changes in anisometropia and age in early childhood.

Our study revealed a higher baseline anisometropia in the myopic group than in the hyperopic group. This concords with the results of a study conducted on elementary school students in Taipei City, Taiwan; the study reported a higher proportion of severe anisometropia in children with myopia than in those with other types of refractive errors^3^. During our study period, the differences in anisometropia by age became less notable in the antimetropic and myopic groups before the age of 6 years, and were similar to that of the hyperopic group after the age of 6 years. Only one study compared children with myopia and hyperopia; it included 30 children with amblyopia who had a mean age of 5 years^8^. The study reported a significant reduction in anisometropia in children with amblyopia and myopia; however, anisometropia in children with amblyopia and hyperopia remained stable. In a study on children with myopic anisometropia, a significant reduction in anisometropia was noted^13^. However, these studies have only compared initial and final SE and have not investigated the changes in anisometropia by age. Our study included a large number of children with anisometropia and demonstrated that anisometropia decreases in children younger than 6 years, and that the changes in anisometropia also became less notable by age in children with myopia or antimetropia. A longitudinal study from Egypt reported a nonsignificant decrease in anisometropia in children with myopia who were 0–8 years old at first visit and were diagnosed with anisometropia of 4.00 D or higher^12^. This nonsignificant result may be because the study did not categorize the participants according to their age. Another reason may be the fundamental disparity in the study population between that study population and ours. The prevalence of myopia, is much higher in Taiwan than in Egypt^16,17^. Moreover, myopia progression extended until school age in Taiwanese children^18^, whereas stable refraction after 4 years of age was reported in the Egyptian study^12^. These factors may have contributed to the disparity in changes in anisometropia between the aforementioned study and ours.

The results of this study’s regression analysis reveal that amblyopia and exotropia were associated with high anisometropia in all three refractive groups. Previous studies have reported that both amblyopia and strabismus were associated with a high anisometropia in children^19,20^. In a study on animals, strabismus was associated with anisometropic development by interfering with the vision-dependent mechanism of ocular growth during the emmetropization process^21^. In our study participants, strabismus and anisometropia were diagnosed at the first few visits; therefore, we cannot establish the sequential relationship between these disorders. Atropine can be used to slow myopia progression^22^ or treat amblyopia^23^, and theoretically, it can interfere with anisometropia. However, in our study, atropine use was not associated with changes in anisometropia. This may be because most children in our cohort had hyperopia or myopia, and half of the children in the antimetropic group did not use atropine. In other words, atropine was not used at all or used in both eyes at the same time in most of our children. During the follow-up period, 11 children did not fit the definition of their initial refractive groups. Among these children, the mean anisometropia increased in the children with initial hyperopia and decreased in the children with myopia or antimetropia. This is in line with our finding that the changes in anisometropia by age were not similar in the different refractive groups.

The limitations of this study should be noted. First, while the hospital-based nature of our study may constrain its applicability to a wider population, the infrequency of anisometropia makes a longitudinal, population-based design impractical in clinical contexts. To our knowledge, this study encompasses the largest number of children compared to similar research efforts, with the children from two separate medical centers in distinct geographic regions. Second, not all children returned to the clinic in a regular pattern due to personal reasons. However, more than two thirds of the children in this study were followed up for > 4 years. Third, regrouping was not performed for children who changed their initial refractive groups during the follow-up period. However, only 7% of the children underwent a group change, and the changes in anisometropia were compatible with that of the initial group. Fourth, some children received cycloplegic refraction with tropicamide rather than atropine, making residual accommodation a concern. We investigated the relative change in refraction between 2 eyes in a child instead of evaluating the absolute value of refraction in each eye, and this design minimized the concern. Furthermore, tropicamide has been commonly used in studies to achieve cycloplegic refraction^24,25^. Despite these limitations, our study demonstrated that the change in anisometropia was most notable in children at younger age, and the change was found in children with myopia or antimetropia, but not hyperopia.

## Conclusion

Our result demonstrated that the correlation between changes in anisometropia and age does not exhibit linearity in the children aged 3–13 years. Anisometropia decreased the children aged < 6 years. This decrease is most notable in children at younger age and it is associated in children with myopia or antimetropia.

## Data Availability

The datasets used and/or analysed during this study are available from the corresponding author on reasonable request.
